# Emerging microfluidic technologies for microbiome research

**DOI:** 10.3389/fmicb.2022.906979

**Published:** 2022-08-16

**Authors:** Yue Yu, Hui Wen, Sihong Li, Haojie Cao, Xuefei Li, Zhixin Ma, Xiaoyi She, Lei Zhou, Shuqiang Huang

**Affiliations:** ^1^CAS Key Laboratory of Quantitative Engineering Biology, Guangdong Provincial Key Laboratory of Synthetic Genomics, Shenzhen Institute of Synthetic Biology, Shenzhen Institutes of Advanced Technology, Chinese Academy of Sciences, Shenzhen, China; ^2^Faculty of Synthetic Biology, Shenzhen Institute of Advanced Technology, Chinese Academy of Sciences, Shenzhen, China; ^3^University of Chinese Academy of Sciences, Beijing, China

**Keywords:** microfluidics, microbiome, microbial cultivation, phenotype screening, technologies integration

## Abstract

The importance of the microbiome is increasingly prominent. For example, the human microbiome has been proven to be strongly associated with health conditions, while the environmental microbiome is recognized to have a profound influence on agriculture and even the global climate. Furthermore, the microbiome can serve as a fascinating reservoir of genes that encode tremendously valuable compounds for industrial and medical applications. In the past decades, various technologies have been developed to better understand and exploit the microbiome. In particular, microfluidics has demonstrated its strength and prominence in the microbiome research. By taking advantage of microfluidic technologies, inherited shortcomings of traditional methods such as low throughput, labor-consuming, and high-cost are being compensated or bypassed. In this review, we will summarize a broad spectrum of microfluidic technologies that have addressed various needs in the field of microbiome research, as well as the achievements that were enabled by the microfluidics (or technological advances). Finally, how microfluidics overcomes the limitations of conventional methods by technology integration will also be discussed.

## Introduction

Microorganisms are found in almost all types of environments on earth. They are shaping our planet in an imperceptible but substantial way. Through playing a critical role in geochemistry and ecosystem, they are also significantly affecting humankind’s life or even future fate (Blaser et al., 2016). The study of microorganisms can trace back to the invention of the first microscope by Leuwenhoek, who is known as the father of microbiology. Since then, until the mid-19th century, the concept that microorganisms exist as single cells was generally accepted. As our understanding of microorganisms gets more profound, it is now certain that microorganisms exist within complex consortiums while species interactions are fundamental to population dynamics and functions (Berg et al., 2020). This conceptual change in the understanding of microorganisms, together with the rapid development of novel DNA sequencing and multi-omics technologies during the past decades, has promoted the thrive of microbiome research in the 21st century.

The term microbiome was firstly proposed by Whipps et al. in 1988 and recently amended as “a characteristic microbial community occupying a reasonably well-defined habitat which has distinct physio-chemical properties” (Berg et al., 2020). The implications of the microbiome on human health and disease had attracted the greatest interest, subsequently come up with the Human Microbiome Project (HMP) since 2007, which now have entered the second stage, with the mission to elucidate the role of the microbiome on the occurrence and development of inflammatory bowel disease (IBD), type 2 diabetes (T2D), and establishment of the nascent microbiome in neonates (Human Microbiome Project Consortium, 2012a,b; Integrative HMP Research Network Consortium, 2014). In addition to the HMP, other projects, which focus on the environmental microbiome and aim to solve more universal challenges such as population growth and climate change, are also flourishing (Singh et al., 2018; De Filippis et al., 2021). The advances in the field of microbiome research will undoubtedly improve the quality of our lives, though there is still a long journey ahead. Before elucidating the complex mechanisms of how the microbiome impacts human health and biosphere homeostasis, as well as further engineering the microbiome for better serving human society, it is essential to have a comprehensive understanding of the microbiome that inhabits diverse ecological niches. Taxonomic compositions and physiological functions are generally considered two of the most critical aspects of the microbiome. Successful characterization of these two profiles would presumably promote our understanding of the microbiome and the complex mechanisms underlying the emergence of disease and ecological disturbance.

Technological advances in sequencing and multi-omics have enhanced our understanding of the microbiome. Specifically, represented by 16S rRNA gene amplicon sequencing and shotgun metagenomic sequencing, culture-independent methods are widely used for microbiome research nowadays (Arnold et al., 2016; Young et al., 2021). These methods enable more accurate profiling of the microbiome composition and structure, because the unculturable microbial majority is also accounted during the analysis. Moreover, the genus to strain-level taxonomic resolution is achievable in a low-cost and high-throughput manner, and physiological functions can be inferred based on gene annotations. However, the limitations of these methods are also apparent (Arnold et al., 2016). For example, DNA extraction and PCR process before 16S rRNA gene amplicon sequencing may introduce bias and errors. The sensitivity of these methods is inadequate for low-abundant members. The precision and accuracy of taxonomic and functional annotation strongly rely on the quality of the reference database. In addition, the complexity of the selective expression of metabolic genes significantly compromised the physiological function assessment results. By simultaneously measuring the gene transcription, protein expression, and metabolic activity, multi-omics technologies partly solved the limitations mentioned above. However, the challenges in interpreting high-dimensional datasets still hamper the wide-range application of multi-omics analysis. To better identify essential species based on sequencing and understand the physiological functions of specific genes, the culture-based approach has received renewed attention in recent years. Successful isolation and cultivation of previous uncultured lineages significantly expanded database capacity (Lagier et al., 2018). By providing a continuous supply of cells, the culture-based approach not only makes reproducible investigations and validations of mechanisms possible, but also enables the implementation of further industrial applications (Lewis et al., 2021). Nevertheless, as one of the traditional methods, the culture-based approach still suffers from inherent shortcomings like low-throughput, labor-intensive, and high-cost.

To overcome the limitations of traditional technologies, microfluidics is one of the new technologies that have shown its prominence in greatly advancing the field of microbiome research. Microfluidics, also known as lab on a chip, is a multidisciplinary field that forces the manipulation of fluids at a typically sub-millimeter scale (Whitesides, 2006). The original intention of microfluidics is to rescale conventional biology and chemistry laboratories onto a square centimeter-level chip for higher throughput and lower cost. It has emerged as a fascinating technology in diverse biological research areas due to its perfect size matching effect with biological samples (Sackmann et al., 2014; Scheler et al., 2019; Hu et al., 2021). In recent years, microfluidics has demonstrated great potential for revolutionizing the microbiome research, and it is believed that its universal application will promote our understanding of the microbiome (Ballard et al., 2018; Zhang et al., 2022). This review will introduce recent developments in applying microfluidics to study the microbiome. The content of this review will cover (1) key microfluidic technologies for microbiome, (2) new species isolation and taxa discovery, and (3) phenotype screening and sorting. The discussion will focus on how microfluidics technologies bypass or overcome the limitations of other culture-independent and culture-based technologies, as well as the combinations with those non-microfluidics technologies.

## Key microfluidic technologies for the microbiome

Among all the microfluidic technologies, droplet microfluidics is the most commonly adopted for diverse microbiome applications. In a microfluidic device, monodispersed water-in-oil droplets are normally generated in a high-throughput manner. These micrometer-size droplets can provide millions of segregated micro-environments for independent cultivation, detection, and handling of microbial cells with distinct biological properties. The advantage of droplet microfluidics also lies in its feasibility of integrating with various devices to monitor phenotypic or genotypic features of captured microbes. Characterization of the microbiome from a population-scale or isolation of interested individuals at the single-cell level is relatively easy to achieve with this technology. According to the technical routes, droplet microfluidics can be categorized as floating droplet approaches and static droplet approaches.

### Floating droplet microfluidics

**Figure 1:**
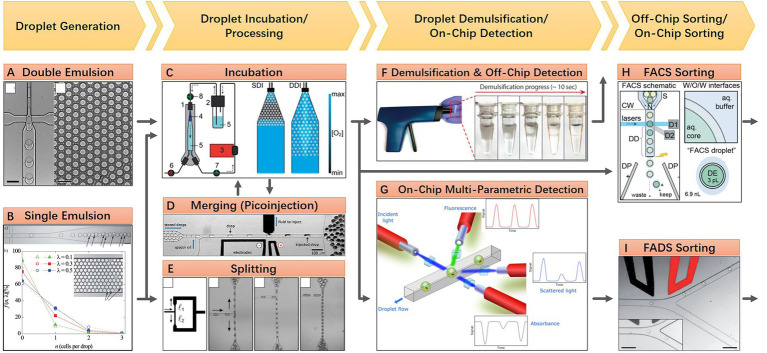
A general workflow of floating droplet microfluidics. **(A)** Generating water-in-oil-in-water double emulsion droplets in a PDMS microfluidic chip, the monodispersed double emulsion droplets are compatible with a commercial flow cytometer (Reprinted from Bauer et al., 2010 with the permission of Royal Society of Chemistry); **(B)** Generating water-in-oil single emulsion droplets in a PDMS microfluidic chip, individual cells are encapsulated during droplet generation and distribution of cells in droplets follows a Poisson distribution. The monodispersed single emulsion droplets are more flexible to be handled and processed (Reprinted from Köster et al., 2008 with the permission of Royal Society of Chemistry); **(C)** A customized off-chip droplet incubator with enhanced oxygen supply (Reprinted from Mahler et al., 2015 with the permission of Royal Society of Chemistry); **(D)** An electrically induced droplet merging method called picoinjection for selective adding new reagents into droplets (Reprinted from Abate et al., 2010 with the permission of National Academy of Sciences); **(E)** A geometry induced droplet splitting method for removing partial contents with desired ratio (Reprinted from Link et al., 2004 with the permission of American Physical Society and the author); **(F)** An electric induced droplet demulsification method for downstream treatments after droplet processing (Reprinted from Karbaschi et al., 2017 with the permission of AIP Publishing); **(G)** On-chip multi-parametric detection after droplet incubation (Reprinted from Hengoju et al., 2020 with the permission of AIP Publishing); **(H)** Off-chip sorting of double emulsion droplets using commensal flow cytometer (Reprinted from Brower et al., 2020 with the permission of Royal Society of Chemistry); **(I)** On-chip sorting of single emulsion droplets using microfluidic sorter (Reprinted from Baret et al., 2009 with the permission of Royal Society of Chemistry).

Floating droplet approaches are featured by their ultra-high-throughput sample processing ability. Its workflow typically includes droplet generation, droplet incubation, direct droplet detection, or pool detection after droplet demulsification. Droplet merging or splitting can be integrated into the workflow if the spatial or temporal environment needs to be regulated. Droplet sorting can be employed when the microbial targets need to be isolated for further investigation. These various droplet manipulation methods constitute a versatile toolbox for researchers to use in combination according to their research purposes. Here, a general workflow of floating droplet microfluidics for microbiome research is shown in Figure 1. For any specific experiment, the first step is always droplet generation. According to the microfluidic chip design and the perfusion velocity of the liquid sample, the droplet generation frequency can range from 10^2^ to 10^4^ Hz, while the droplet size can range from tens to hundreds of microns (Bauer et al., 2010). In accompanied by droplet generation, microbial cells are encapsulated in droplets, either in the form of single-cell or multiple cells. And the cell number in a droplet can be estimated and well-controlled according to Poisson distribution (Köster et al., 2008). This stochastic confinement approach has been used to simulate the natural compartmentation phenomenon of the soil microbiota, and the droplets are analogous to the microporous structure of soil (Cao et al., 2017). For the second step, droplets are usually collected in a commercial or customized container for off-chip incubation. During the incubation process, gas and temperature conditions are precisely controlled, permitting microbial cells to proliferate (Mahler et al., 2015). For the third step, phenotypes or genotypes of encapsulated cells can be detected through either off-chip or on-chip approaches. For off-chip strategies, the droplets can be detected using commercial flow cytometry (Brower et al., 2020), or the droplets can be demulsified for conventional microbial testing (Karbaschi et al., 2017). For on-chip strategies, a monolayer of droplets can be either examined under microscopy in a microfluidic chamber whose height is smaller than droplet diameter, or droplets can be reinjected into and then flow through another specific microfluidic chip for photoelectric detection (Hengoju et al., 2020). Isolation of microbial targets can be synchronously accomplished by implementing droplet sorting, and the targeted cells can be retrieved after demulsifying the sorted droplets (Baret et al., 2009). Besides these three main steps, other optional steps can be added to the workflow to meet different experimental needs. For instance, droplet splitting and merging operations can be executed to remove partial content or add a new substance if needed (Link et al., 2004; Abate et al., 2010). The removed or added quantity can be accurately controlled by finely turning the corresponding parameters of the microfluidic chip according to the droplet size. Being developed for more than 20 years, the arsenal of floating droplet microfluidic toolbox has been dramatically extended. For each of the up-mentioned droplet manipulation methods, there are numerous technical solutions to meet the demand in different application scenarios. As those solutions have been elaborated in previously published review articles, they will not be introduced again in this review (Kaminski et al., 2016; Matula et al., 2020; Hu et al., 2021).

### Static droplet microfluidics

**Figure 2:**
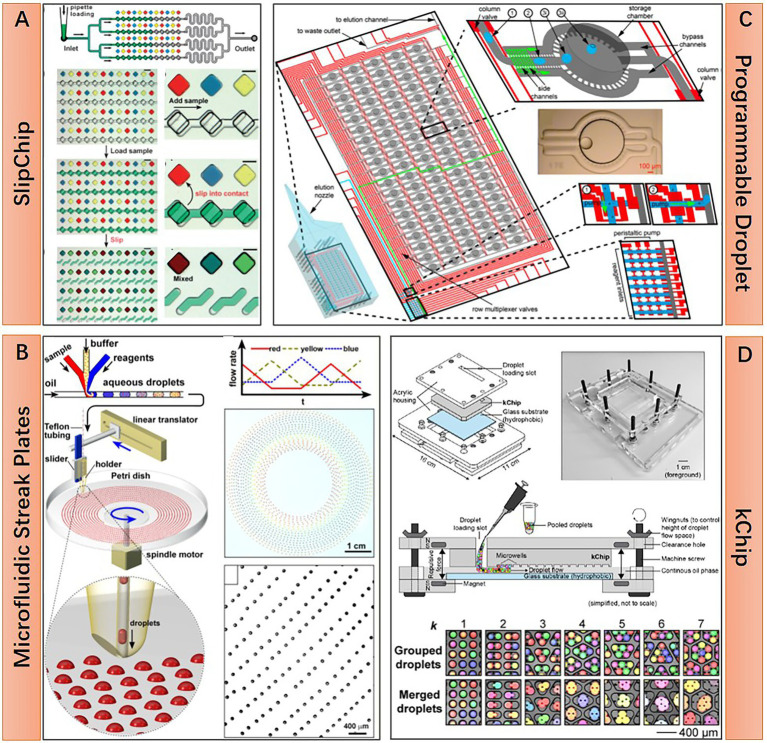
Structural features and working principle of diverse static droplet microfluidic approaches. **(A)** SlipChip approach, preloaded 48 droplet samples can be repeatedly manipulated in parallel by two simple steps operation, sample loading and chip slipping (Reprinted from Du et al., 2009 with the permission of Royal Society of Chemistry); **(B)** Microfluidic streak plates (MSP) approach, on-chip generated droplets are streaked on Petri dish to form sessile droplet array, one Petri dish can hold 1,600 droplets with 6.2 nl volume (Reprinted from Jiang et al., 2016 with the permission of American Society for Microbiology); **(C)** Programmable droplet microfluidic approach, in each of the 95 storage chambers, droplet immobilization and coalescence are realized by flow-controlled wetting, flow control is realized by programmable quake valve operations (Reprinted from Leung et al., 2012 with the permission of National Academy of Sciences); and **(D)** kChip approach, pre-generated droplets are loaded into the kChip by simply pipetting and washing, *k* represents the number of droplets that a microwell can hold, droplets in the same microwell will merge to a larger one after applying electric field, the droplet capacity of a kChip ranging from 430 to 2,000 per cm^2^, depending on the *k* value (Reprinted from Kehe et al., 2019 with the permission of National Academy of Sciences).

Static droplet approaches are featured by their planar arrangement of position-fixed droplets. This droplet layout enabled the real-time monitoring of each droplet under microscopy for an extended period, as well as indexing and on-demand individual recovery of targeted microbes in droplets at any time. As water-in-oil droplets, static droplets are either confined in chambers of microfluidic devices or anchored in the hydrophilic surface under the surface-wetting effect (Gao et al., 2013). Some representative static droplet approaches are shown in Figure 2. SlipChip and Microfluidic Streak Plates (MSP) are two representatives of those droplet immobilization approaches. SlipChip is mainly constructed of two pieces of glass plates with concave microstructures, like wells or trenches, on the surface. Enclosed chambers or channels are formed when these two plates are assembled and aligned. Fluorinated oil like FC-40 is filled between the two plates. This oil acts as a lubricant and the continuous phase of the droplet. Aqueous droplets are immobilized in the enclosed chambers. SlipChip functions *via* the relative sliding of the two plates. Programmed sliding results in linking, disconnecting, combining, or disassembling the microstructures (Figure 2A). Parallel handling of droplets is achieved based on these sliding operations (Du et al., 2009). The enclosed microenvironment in SlipChip is particularly suitable for culturing anaerobic microorganisms. For example, Ma et al. culture anaerobic gut microbes within a Replica-SlipChip. This variant of SlipChip design can split each microcolony into two identical copies, one for genetic assay and the other for targeted recovery based on the assay results (Ma et al., 2014a). In contrast, MSP is easier to implement and scale up. MSP device is usually constructed with three main components: a microfluidic chip for generating droplets, a petri dish for carrying droplets, and a writing tip for transporting droplets from a microfluidic chip to a petri dish with the manner of streaking (Figure 2B). The surface of the petri dish is modified to be hydrophilic and then covered with a layer of oil which is the same as the droplet continuous phase. As long as the droplet contacts the surface during manual or automated streaking, it will be fixed in a specific position and then covered by the preloaded oil due to the surface-wetting effect (Jiang et al., 2016). Isolation of targeted microcolonies in the droplets can be realized through manual picking or a semi-automatic droplet picker (Hu et al., 2020). Until now, the MSP approach has achieved a series of successes in cultivating and isolating fastidious environmental microorganisms. Besides SlipChip and MSP, many other static droplet approaches have also been reported. Various microstructures that can capture and immobilize droplets are adopted in these works. Leung et al. present a programmable droplet-based microfluidic device for physiological and genomic analysis of microbial single cells or consortia (Leung et al., 2012). This device takes full advantage of the microfluidic large-scale integration for the complex operation of each static droplet (Thorsen et al., 2002). Diverse functions like single-cell sorting, cultivation, whole genome amplification, and sequencing can be integrated into a single microfluidic chip if necessary (Figure 2C). In another work, Kehe et al. report a kChip device that shows a remarkable ability to characterize microbial phenotypes and analyze microbial community ecology (Kehe et al., 2019). The kChip contains tens of thousands of microwells for capturing droplets. The size and geometry of each microwell are designed to hold a specific number of droplets. In this manner, droplets in the same microwell are grouped. In the workflow of the kChip experiment, a droplet library containing various microbial cells or chemical factors is firstly generated, followed by the random grouping of these droplets in the microwells. Subsequently, an alternating-current electric field is applied to the kChip to merge the grouped droplets, resulting in the parallel establishment of microbial communities (Figure 2D).

A usage comparison of floating droplets and static droplets approaches for processing different microbiome sample types and meeting different application purposes is illustrated in Table 1.

**Table 1:** A usage comparison of floating droplets and static droplets approaches.

	**Floating droplets**	**Static droplets**			
		**SlipChip**	**MSP**	**kChip**	**Programmable microfluidic reaction array**
Sample types (Anaerobic cultures are marked by *)	Bacterial pure culture. Environmental microbiota sampled from soil and pond water. Human microbiota sampled from oral, gastrointestinal tract*, and feces*. Animal microbiota sampled from bear oral.	Bacterial pure culture*. Environmental microbiota sampled from soil. Human microbiota sampled from gastrointestinal tract*.	Bacterial pure culture. Environmental microbiota sampled from soil and deep sea sediment. Animal microbiota sampled termite gastrointestinal tract*.	Bacterial pure culture. Environmental microbiota sampled from soil.	Bacterial pure culture. Environmental microbiota sampled from marine enrichment culture, deep sea sediment. Human microbiota sampled from oral.
**Typical workflow**	Droplet encapsulation. On/Off-chip incubation. Addition of bacterial cells or functional compounds. Growth detection and on/off-chip droplet sorting. Demulsification of droplets to release bacterial cells. Off chip expanded cultivation and isolation. Taxonomy classification by sequencing. Metabolites analysis by using LC–MS.	Loading of reagents and bacterial cells. Manipulation of the liquid in droplets by sliding the chip. Culture and on chip PCR. Off chip expanded cultivation and isolation. Taxonomy classification by sequencing.	Droplet encapsulation. Manual or automatic streaking droplets on Petri dish. Culture. Manual or semi-automatic droplets collection. Expanded cultivation. Taxonomy classification by sequencing.	Encapsulation of microbial cells in color-coded droplets. Pool droplets and load into kChip to form groups. Merge droplet to generate parallel synthetic communities. Tracking the communities’ phenotypes *via* optical assays.	Droplet encapsulation and loading into a separate storage chamber. On chip incubation and imaging. On chip PCR and WGA. Sample recovery and off-chip sequencing.
**Applications**	New taxa discovery and isolation. Study of cell–cell interactions. Screening for antibiotic-resistant species. Mining of microbiome resources.	Chemotaxis-based cell sorting. Genetically targeted isolation of interested taxa.	Antimicrobial susceptibility testing. Recovering rare microbial taxa from various environments.	Construction and screening of microbial communities.	Microbes cultivation. Taxonomy classification.

Anaerobic cultures are marked by *, which means that the droplet microdevice was manipulated under anaerobic environment.

### Microfluidic microwell array

**Figure 3:**
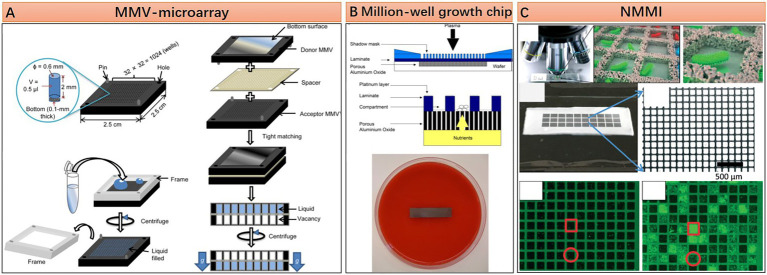
Structural features and working principle of diverse microfluidic microwell array approaches. **(A)** An microarray with manageable volumes (MMV)-microarray with 2.5 by 2.5 cm dimension contains 1,024 wells of 0.5 μl volume each, manipulation of liquid in each microwell is based on well-to-well transferring with the assistance of silicone or urethane spacer (Reprinted from Sharma et al., 2014 with the permission of Springer Nature and the author); **(B)** Million-well growth chip, for an 8 × 36 mm chip, there are 180,000 compartments with 20 by 20 μm dimension each, porous aluminum oxide located under the compartments for nutrition and metabolic waste diffusion (Reprinted from Ingham et al., 2007 with the permission of National Academy of Sciences); and **(C)** Nanoporous microscale microbial incubators (NMMI), the wall is constructed by HEMA–EDMA hydrogel, the pore size is small enough to allow diffusion of metabolites but not the cells (Reprinted from Ge et al., 2016 with the permission of Royal Society of Chemistry).

In contrast with the enclosed system of droplet microfluidics, microfluidic microwell array approaches possess some unique advantages for microbiome research owing to their open geometry feature. With this geometry, reagents or cells can be added to or removed easier, thus facilitating some complex tasks requiring multi-step parallel processing. Several representative microfluidic microwell array approaches are shown in Figure 3. The microarray with manageable volumes (MMV) chip invented by Sharma et al. is designed for performing genome profiling (GP) analysis on the oral microbiome. The experiment pipeline consists of multiple steps, including single-cell isolation, single-cell DNA extraction, single-cell random PCR, and sample transferring for subsequent micro-temperature gradient gel electrophoresis (μTGGE). Parallel pipette-free handling of nanoliter volumes enabled by the MMV chip significantly reduced the operation time and facilitated the comprehensive analysis of oral microbiome with complex composition (Figure 3A; Sharma et al., 2014). The open geometry feature of microfluidic microwell array devices can also provide continuous nutrients supplying and metabolic wastes expelling during microbial cell cultivation, which guarantees a better culture condition for fastidious microbes. This point is well demonstrated by Ingham et al. and their million-well growth chip. The chip comprises top chambers for single-cell culture and a bottom porous membrane for nutrient diffusion. Excessive nutrients are supplied from the agar plate under the chip (Figure 3B). Culturing the environmental microbiome in a million-well growth chip enables the isolation of several previously uncultured species (Ingham et al., 2007). The microwell array device can also be transferred to the original location of the environmental microbiota for *in situ* cultivation. This approach is considered effective in resuscitating the “uncultivable” majorities in the microbiome (Nichols et al., 2010). Moreover, the microwell array fabricated by nanoporous hydrogel enables the intercellular cross-feeding and signaling, which has been proven critical for the growth of some symbiotic microorganisms (Figure 3C; Kaeberlein et al., 2002). Specifically, Ge et al. show that such a device can be used to study quorum sensing or even elucidate the growth-dependent relationship within a population (Ge et al., 2016). It is worth mentioning that Xu et al. demonstrate that even a piece of hydrogel without any pattern can act as a microfluidic microwell array for complex single-cell sequencing experiments, and they name this virtual microfluidics (Xu et al., 2016). Their work provides a lot of inspiration. That is, microfluidic technologies should not be restrained in the scope of microfabrication and fluidic manipulation. It can appear in any form as long as it can make great use in any research field.

## Applications

### New taxa discovery and isolation

Lots of new bacteria and archaea species have been identified due to the rapid development of metagenomic technology. By contrast, the hitherto isolated and cultured species are estimated to be less than 40 % of human gut microbiota and no more than 1 % of environmental microbiota (Lloyd et al., 2018; Liu et al., 2021). Obtaining the pure culture of a species from poorly studied taxa without any cultured representative is generally considered hugely important, as this will facilitate a series of meaningful *in vitro* studies. For example, gene functions or physiological traits inferred from metagenomic information can be tested experimentally. Even the gene regulations or metabolic pathways that are hardly inferred from metagenomic data can also be further validated. Additionally, a reference database derived from a complete collection of cultured species will significantly enhance our ability to interpret multi-omics datasets, thereby improving our understanding of how the microbiome interacts with its ecological niches. These urgent demands of cultured lineages lead to a new round of efforts to culture the previously considered unculturable microorganisms. To satisfy the rigorous growth requirements of many fastidious species, traditional cultivation methods usually involve testing massive media combinations and physicochemical conditions, accompanied by time-consuming and laborious isolation methods like dilution-to-extinction or colony picking. To achieve better performance, new techniques mainly resort to two strategies, increasing the throughput (high-throughput isolation) or targeting the desired taxonomic groups (targeted isolation; Lewis et al., 2021). One example of high-throughput isolation is the culturomics technique, which integrates MALDI-TOF mass spectrometry and 16S rRNA sequencing into the workflow to increase the detection and isolation efficiency (Lagier et al., 2018). Targeted isolation is represented by a technique called reverse genomics, in which genome-informed antibodies were engineered to label the desired species for sorting (Cross et al., 2019).

Among diverse techniques, microfluidics manifests itself as a powerful tool to discover and isolate new taxa. This is attributed to its technical flexibility, which facilitates it to achieve the aforementioned high-throughput and targeted isolation purposes simultaneously. In microfluidic devices, compartment, monitoring, detection, and fine manipulation of a single cell is easily achievable through a scalable and high-throughput manner. Processing microbiota *via* the single-cell way eliminates the competition between species, and prevents fast-growing species from outcompeting slow-growing species or dormant cells, thus increasing the chance of isolating those fastidious or ultra-low-abundance species. Droplet microfluidics proves to be an effective way for compartmenting a microbial community. The compartmentation generates hundreds of thousands to millions of subpopulations with single or few cells. Researches have shown that dispersing and culturing human gut microbiota in droplets leads to the detection of some taxa that were missed by metagenomic survey (Villa et al., 2020), or even the discovery of previously uncultivated new taxa (Tan et al., 2020). In a study of the termite gut microbiome, a series of previously uncultivable bacterial taxa are isolated using the MSP-based culture method. Meanwhile, several taxa, which have never been detected in the termite gut microbiome by metagenomic methods, are successfully cultured. Several isolated strains were classified into novel species, or even novel genera (Zhou et al., 2019). These results indicate that merely a single high-throughput compartmentation operation is beneficial for discovering new taxa from a microbiota.

Once the microbial cells are individually compartmentalized, the entire microfluidic device can be transferred to the original habitats of the microbiome for *in situ* cultivation. This strategy thoroughly considers the important effects of local environmental factors on cultivating fastidious microbes. These factors may be elusively chemical or biological compounds that are extremely difficult to be identified and reproduce in the laboratory. Besides, the poor nutrient supply of most natural habitats is more profitable for the growth of oligotrophic microorganisms, and the overgrowth of dominant species thus can be avoided. One successful case of the microfluidic *in situ* cultivation strategy is the Ichip. The Ichip comprises 384 separated chambers with 1 mm diameter for loading single cells. The geometry of each chamber is a cylindrical through-hole, which is sealed by two porous membranes from each side. This configuration enables the substance exchange of the encapsulated cells with their surrounding environment. Microorganisms encapsulated in Ichips are returned to their native habitats for long-term incubation. Compared with artificial culture conditions like on agar plates, the *in situ* cultivation in Ichip enables increased recovery of new taxa from either the seawater or soil microbiome (Nichols et al., 2010). Moreover, a novel depsipeptide antibiotic without detectable resistance is discovered when screening soil microbiome from a grassy field (Ling et al., 2015). In an earlier study, Zengler et al. demonstrate a method to stimulate the original environment of marine microbes in the lab. The microbial cells are encapsulated in gel microdroplets (GMDs), and the GMDs were then collected in a growth column. Filter-sterilized seawater obtained from the original habitat is used as a culture medium to flow through the growth column. Compared to the *in situ* cultivation approach, the experiment throughput is improved dramatically (Zengler et al., 2002). Most recently, a microbe domestication pod (MD Pod) was invented (Alkayyali et al., 2021). This method combines the advantages of both GMDs and *in situ* cultivation strategies, significantly increases the throughput, and simplifies the operation. The further performance of such kinds of *in situ* approaches is anticipated.

Beyond the aforementioned non-targeted cultivation and isolation approaches, microfluidics is also highlighted by its ability to integrate with versatile monitoring, detection, cultivation, and automation methods for targeted isolation of interested taxa without sacrificing throughput. Currently reported strategies include integrating microscopic imaging, fluorescence or turbidity detection, and genetic assay into a microfluidic workflow.

#### Microscopic imaging enabled targeted isolation

Due to the ordered 2D arrangement of sessile droplets on the surface of commercial Petri dish, the MSP technique is inherently compatible with microscopic imaging. Physiological traits like cell density and biofilm formation can be characterized when monitoring the microbial cells in MSP droplets under a bright field imaging model. The utility of this approach has been shown in a study for targeted isolating core taxa that can degrade environmental pollutant fluoranthene from a pre-enriched soil microbiota. Four strains of the genus *Mycobacterium* with a slow growth rate are isolated, and these strains exhibit a degradation rate of 99.5%. More notably, a strain belonging to the genus *Blastococcus*, which has not been detected by 16S rRNA gene amplicon sequencing of the original sample, is also isolated. And the fluoranthene degradation rate of this strain reached up to 100%. In contrast, no strain isolated from agar plates with the traditional method exceeds 12.7% (Jiang et al., 2016). An upgraded version of the MSP culture method has exhibited improved performance in enriching rare bacterial species from deep-sea surface sediments. This is achieved by excluding droplets with fast-growing dominant strains with the help of microscopic morphological analysis. Those species with less than 0.01% relative abundance in original samples constitute more than 90% of the population. Besides, 16S rRNA gene amplicon sequencing datasets analysis shows that species diversity, richness, and evenness are significantly higher than in the agar plate cultured pool. Exclusive operational taxonomic units (OTUs) in MSP cultivated pool are three times larger, which is 353 vs. 102 OTUs. Moreover, 15 presumable and three identified novel species are obtained through morphological analysis aided monoclonal picking (Hu et al., 2020).

**Figure 4:**
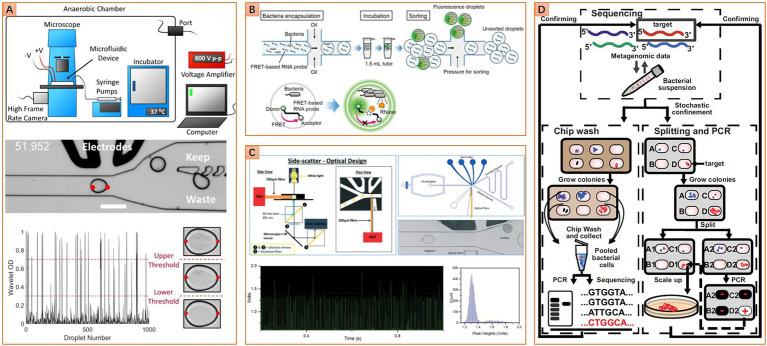
Diverse microfluidic approaches for targeted isolation of interested taxa. **(A)** Image-based approach, image analysis algorithm is used to distinguish droplets with different cell densities (Reprinted from Watterson et al., 2020 with the permission of eLife Sciences Publications Ltd.); **(B)** Fluorescence-based approach, fluorescence resonance energy transfer-based (FRET-based) RNA probe is used to detect metabolic activity in droplets, other kinds of probe like the fluorogenic substrate or resazurin and their derivatives can be set as alternatives (Reprinted from Ota et al., 2019 with the permission of PLOS publishing); **(C)** Turbidity-based approach, variation of droplet turbidity caused by cell proliferation is detected *via* scattered light signal (Reprinted from Liu et al., 2016 with the permission of Royal Society of Chemistry); **(D)** Gene-targeted cultivation and isolation, PCR was carried out on one replica of the SlipChip to locate the target on another replica of the SlipChip (Reprinted from Ma et al., 2014b with the permission of National Academy of Sciences).

Bright field microscopic imaging can also be integrated into the workflow of floating droplet microfluidics. This strategy uses a high-speed camera to continuously acquire images of a succession of droplets flowing through a microchannel. Images are transferred to a computer for real-time analysis. Once a droplet containing the targeted microbe is recognized, it will be recycled *via* droplet sorting (Figure 4A). Works related to this strategy were firstly presented by Zang et al., who successfully identified *Actinobacteria* mycelia and sorted out droplets containing germinated *Actinobacteria* spores (Zang et al., 2013). Most recently, Watterson et al. (2020) improve this technique to recognize the bacterial colony density in droplets. Droplets containing slow-growing human gut microbes were sorted, and the obtained population includes a higher ratio of rare taxa than the original sample (Watterson et al., 2020). Compared to the MSP technique, bright field microscopic imaging-based droplet sorting possesses a much higher droplet screening throughput. When combined with metagenomic sequencing, the chance of discovering new taxa can be increased significantly. However, the sorted droplets should be pooled and demulsified for recycling cells. And those mixed cells need further inoculation and purification if obtaining pure isolates is the final objective. Unless bypassing the droplet pooling step by directly distributing the sorted droplets into individual wells (Cole et al., 2017), those additional operations will significantly compromise the efficiency of isolating new taxa. Moreover, the bright field microscopic imaging-based strategy has been used to distinguish the difference in color or shape between single microalgae cells (Girault et al., 2017; Kraus et al., 2021). Although this in-droplet single-cell analysis function is currently limited to large cells, further improvement of imaging resolution and image processing ability may transform this technique into in-droplet single microbial cell analysis.

Integrating with fluorescence microscopic imaging further extends the application fields of microfluidics, such as identification and isolation of microbes with different nutrition preferences. One such case is presented by Ingham et al. (2007). They developed a “million-well growth chip” based on a microfluidic microwell array strategy for recovering oligotrophic microorganisms. The chip comprises top microchambers for single-cell compartmenting and a bottom nanoporous membrane for mechanical support and nutrient diffusion. Combined with the supply of fluorogenic organophosphate compound and fluorescence microscopy imaging-based growth monitoring, six previously uncultured species are successfully isolated from Rhine water sample, and at least one may belong to an uncultured genus (Ingham et al., 2007).

#### Fluorescence or turbidity detection enabled targeted isolation

Instead of monitoring the fluorescence signal in a microwell array with fluorescence microscopy, floating droplet microfluidics provides a more efficient way for high-throughput fluorescence detection. This is usually accomplished with the assistance of microfluidic fluorescence-activated droplet sorting (FADS) or flow cytometry-based droplet sorting technique (Baret et al., 2009; Brower et al., 2020). A universal metabolic indicator like resazurin can be used to detect the growth of microbes in droplets. Living cells can reduce the non-fluorescent resazurin to green fluorescent resorufin. The fluorescence intensity is usually correlated with the metabolic activity of the cell. When culturing single microbial cells in droplets with a specific medium formula, species that can utilize the nutrition in the medium can be targeted and isolated by detecting the fluorescence intensity of the droplet.

However, the resorufin is not an ideal fluorescence indicator for the water-in-oil droplet system, because it can leak into the oil phase and then transfer between water droplets. For applications that need long-term cell culture, this leakage may cause a severe false positive problem. To solve this problem, researchers are pursuing alternative indicators with better droplet retention performance. A derivative of resorufin, dodecylresorufin, has improved retention performance in 24 h droplet incubation (Scheler et al., 2016). However, most microbiome samples require more prolonged cultivation duration. This is especially true for the environmental microbiome, for which the culturing period is usually up to a month. Based on this consideration, a fluorescent nucleic acid probe (FNAP) has been developed to detect the growth of environmental microbes in droplets. The fluorescence of FNAP is quenched by the fluorescence resonance energy transfer effect. Living cells can release ribonuclease, such as RNase, which will cleave the FNAP (Figure 4B). Once the FNAP is cleaved, the fluorescence intensity will increase significantly. It has been shown that FNAP works well for evaluating the growth dynamics of water or soil microbiota in the droplet (Ota et al., 2019; Saito et al., 2021). However, considering that the performance of FNAP will be significantly affected by a cell’s ability to express and release ribonuclease, the feasibility of detecting live microbes under diverse metabolic conditions still needs more testing. In particular, when examining a population that contains bacterial cells belonging to different taxa, the detection bias should be evaluated carefully.

Apart from detecting overall metabolic activity, the droplet-based fluorescence assay system may also be designed to target microbes that can produce specific metabolites. For example, utilizing a metabolite oxidase to oxidize the target metabolite specifically, and then detect the concentration of the by-product hydrogen peroxide. The fluorescence sensing system coupled with horseradish peroxidase can be used to quantify the hydrogen peroxide concentration. This approach has been exploited to identify xylose-overconsuming *Saccharomyces cerevisiae* cells from a population composed of the same species (Wang et al., 2014). It is promising to apply this technique to microbiome samples for targeted isolation of microbial cells with specific metabolic activities, but further technical modifications are necessary.

For fluorescence-based bacterial growth monitoring in the droplet, unpredictable effects of the fluorescence probes on cell physiology are inevitable. Therefore, label-free growth monitoring strategies are more favorable due to low interference to cells. Turbidity-based bacterial growth monitoring in the droplet is typical of such strategies. The droplets’ light absorbance and scattering properties will change as the change of inside bacterial cell density. Specifically, the growth of microbes in droplets leads to an increase in cell density. Higher cell density in droplets resulted in higher light absorbance and scattering, which can be detected by a photoelectric sensor in a high-throughput manner. As this kind of label-free optical signal typically has lower signal strength and signal-to-noise ratio compared to a fluorescence signal, an optical fiber should be integrated into the microfluidic chip to get close to the droplets for collecting signals more efficiently. This setup slightly increases the fabrication complexity of the microfluidic device (Figure 4C). However, the speed of droplet turbidity-based detection can be comparable with droplet fluorescence detection due to a similar photoelectric signal detection principle, which is significantly higher than another label-free monitoring strategy, the bright field microscopic imaging. Currently, this approach has been proven to robustly detect the growth of more than 12 bacterial species with different cell morphologies (Liu et al., 2016; Hengoju et al., 2020; Pacocha et al., 2021). Obviously, it holds great promise to be applied to monitoring the growth of each microbial member in a complex community.

#### Genetic assay-enabled targeted isolation

Unlike the aforementioned metabolic-based targeted isolation, Ma et al. acquire impressive achievements in targeted isolation of HMP’s most wanted taxa by using a gene-targeted microfluidic method that integrated SlipChip with genetic assays. In this method, the workflow starts with identifying the existence of the interested taxa in the microbiome by 16S rRNA gene amplicon sequencing, followed by optimizing the incubation condition for maximizing the relative abundance of interested taxa, and finally ends with addressing and recovering targeted strains from one SlipChip replica according to the PCR results from another replica (Figure 4D). One strain that may belong to a previously unidentified genus of the family *Ruminococcaceae* is isolated from human gut microbiota for the first time (Ma et al., 2014b). This approach is somewhat like the reverse genomics-based targeted isolation method, but it adopts a DNA targeting strategy instead of an antibody labeling strategy. The DNA targeting strategy bypasses the complex antibody designing process, and it can target more general cell traits than the antibody labeling strategy. However, the weakness of low throughput is hampering its broader applications. Microfluidic genetic assay-enabled targeted isolation may have broad application prospects in microbiome researches if the throughput problem is overcome in the future.

A brief summary of different microfluidic strategies for non-targeted and targeted taxa discovery and isolation approaches is illustrated in Table 2.

**Table 2:** A brief summary of different microfluidic strategies for non-targeted and targeted taxa discovery and isolation approaches.

	**Approaches**	**Microfluidic strategies**	**Sample types**	**Targeted characteristic**
**Non-targeted**	*In situ*	Ichip; MD Pod	Soil microbiota; Three marine bacteria	None
	Laboratory	Floating droplets; MSP	human gut microbiota; Termite gut microbiota	None
**Targeted**	Microscopic imaging	MSP; Floating droplets; Million-well growth chip	Soil microbiota; Marine microbiota; Human gut microbiota	Cell number change caused by growth
	Optoelectronics	Floating droplets	Soil microbiota; Pure culture of more than twelve bacterial species	Fluorescence change caused by growth; Turbidity change caused by growth
	Genetic assay	SlipChip	Human gut microbiota	16S rRNA gene of a specific genus

### Mining of microbiome resources

Microorganisms on our planet are a fascinating reservoir containing tremendous resources beneficial to humankind. Since ancient times, people have unintentionally exploited microorganisms for life and production activities, such as food and alcohol fermentation. Intentional mining of microorganisms with useful functions was followed by the fast development of microbiology, represented by the milestone success of searching for natural antibiotics. Despite the rapid evolving of multiple sequencing technologies for functional gene assessment, phenotype screening of cultured microorganisms is still the most reliable and broadly adopted approach. However, most microorganisms are still unculturable, either for the environment, animal, or human microbiome. Accompanied by the excessive exploration of culturable microbes, the effectiveness of traditional culture-based phenotype screening methods is weakening because they cannot examine enough amount of colony for new taxa. The appearance of microfluidic technology greatly compensates for these deficiencies. As mentioned in the previous section, microfluidics provides microbiologists a convenient avenue to access the uncultured majority in the microbiome. In this section, how microfluidic technologies facilitate the high-throughput mining of microbiome resources will be discussed in detail.

#### Antibiotic molecules

**Figure 5:**
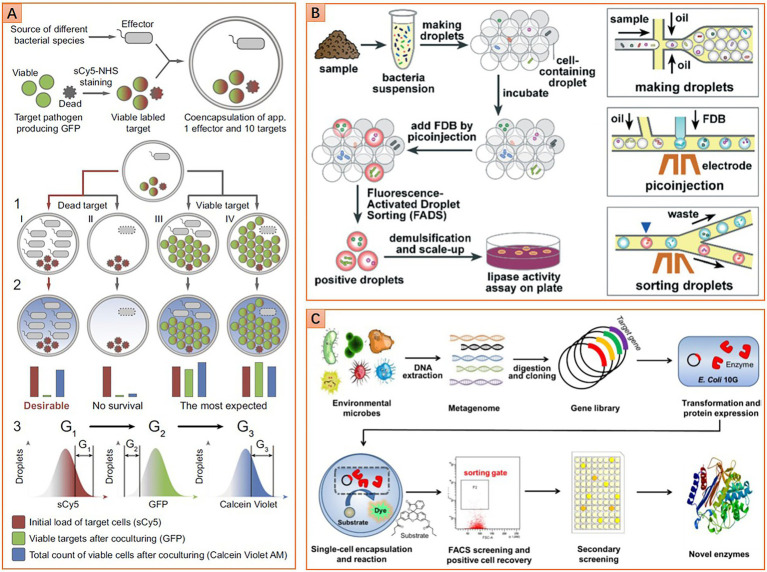
Diverse microfluidic approaches for phenotype screening and sorting. **(A)** Screening microbes with antimicrobial ability, double emulsion droplets are detected and sorted by flow cytometer (Reprinted from Terekhov et al., 2017 with the permission of National Academy of Sciences); **(B)** Screening lipolytic microorganisms based on fluorescence-activated droplet sorting (FADS) method (Reprinted from Qiao et al., 2018 with the permission of Royal Society of Chemistry); and **(C)** Screening esterase genes from the metagenomic library based on a flow cytometric droplet sorting method (Reprinted from Ma et al., 2021 with the permission of John Wiley and Sons).

The microbiome is a reservoir of innumerable genes encoding the synthesis of various antibiotic molecules. The environmental microbiome is the most exploited due to a series of successes in finding natural antibiotics since the discovery of penicillin. However, researchers are encountering bottlenecks in finding new antibiotic molecules from nature after long-term overexploitation of the global environmental microbiome. The success of Ichip proposed the possibility of finding new antibiotics by isolating previously uncultured taxa (Ling et al., 2015). Nevertheless, the randomly cultivated strains with unknown potentials need to be examined individually, which is time-consuming and labor-intensive. Some recent works have shown the potential utility of microfluidics for ultra-high-throughput screening antibiotic producers from a microbiota. These approaches use fluorescent pathogenic cells as reporters to sense antibiotics. These cells are either injected into droplets containing a to-be-screened isogeneic microcolony, or they are co-encapsulated with a to-be-screened single cell into a droplet. Strains with antipathogenic ability will inhibit pathogenic cells’ growth, or even kill those cells, thereby decreasing droplets’ fluorescence intensity. On the contrary, pathogenic cells will grow normally or overwhelm the cells that do not have the antipathogenic ability, resulting in an increment of droplet fluorescence intensity. Thus, the antipathogenic activity can be determined by detecting droplets’ fluorescence intensity (Figure 5A). Sorting of droplets within a specific fluorescence intensity range can be realized by using either FADS or flow cytometry-based droplet sorting. Based on this approach, Mahler et al. successfully isolate a strain (closely related to *Bacillus tequilensis*) that displays strong and broad spectral antimicrobial activity from brown earth soil microbiota. Further research demonstrates that this strain can synthesize five kinds of antimicrobial compounds. Two compounds belong to bacillaene A and B, and the other three belong to the surfactin category (Mahler et al., 2018, 2021). Similarly, Terekhov et al. isolate an uncommon strain of *Pseudomonas aeruginosa* from human oral microbiota. This strain displays a remarkable eradication effect on *Staphylococcus aureus*. Pyocyanin, phenazine-1-carboxylic acid, and heptyl-4-hydroxyquinoline are identified as the primary antimicrobial compounds (Terekhov et al., 2017). In subsequent work, a strain of *Bacillus pumilus* is isolated from Siberian bear oral microbiota. This strain displays antibacterial activity against *Staphylococcus aureus*, and amicoumacin (Ami) is identified as the primary antimicrobial compound (Terekhov et al., 2018). Although these microbial species and antipathogenic compounds are all previously reported, the potential of mining new antibiotic molecules from the microbiome using microfluidics has been proven.

#### Industrial enzymes

The microbiome is also a reservoir that contains countless genes for synthesizing valuable enzymes of industrial importance. Some enzymes may hold great promise for solving increasingly serious social problems, such as energy crisis and environmental pollution. For instance, alcohol derived from cellulosic biomass is considered one of the ideal green energies to substitute traditional fossil fuels. Biocatalysis of cellulose is a practical and eco-friendly approach to producing alcohol, where cellulases with high catalytic performance are imperative to be discovered or evolved. Microfluidics has shown great potential for screening such enzymes (Baret et al., 2009). A general screening workflow is shown in Figure 5B. The superiority of microfluidics in screening cellulases has been demonstrated by Najah et al. (2014). They enrich cellulolytic bacteria from the soil microbiota sampled from wheat stubble. Microbial cells are then individually encapsulated in droplets along with fluorogenic cellobiohydrolase substance, hydrolysis of the substance results in the release of fluorescence molecular. Thus, sorting droplets with high fluorescence intensity enables the recovery of clones with potent high cellulolytic activity. Further analysis shows that cellobiohydrolase activity of the droplet-sorted bacterial population is 17 times higher than that of the agar plate enriched bacterial population (Najah et al., 2014). It is noteworthy that the enriched population also exhibits an elevated endoglucanase activity, although the sorting is not based on the activity of this kind of enzyme. Hydrolases like lipase and esterase are another class of industrial enzymes with broad applications in pharmaceutical, food fermentation, petroleum biocatalysis, etc. To screen lipase, Qiao et al. (2018) use fluorescein dibutyrate as a substrate and indicator to indicate the enzymatic activity. After droplet sorting and following agar plate culturing, 11 species of seven genera with lipase activity are identified from the environmental microbiota. One strain belongs to *Serratia marcescens* with outstanding lipase activity shows great potential for further applications (Qiao et al., 2018).

Another class of enzymes with important industrial value is those capable of degrading organic pollutants for environmental remediation. For example, the pollution caused by ubiquitous plastics, polyethylene terephthalate (PET), is turning increasingly severe. Biodegradation is a favorable way to deal with PET waste. Most recently, an effort to screen PETase with microfluidics from the environmental microbiota was made by Qiao et al., and 17 potential PET-degrading strains belonging to eight genera were isolated from wastewater samples of a PET textile mill. Meanwhile, two PETase belonging to the carboxylesterase and dienelactone hydrolase family are also identified from these strains. They exhibited the known highest degrading activity against Bis(2-Hydroxyethyl) terephthalate (Qiao et al., 2021). Imidazolinone compounds are worldwide popular herbicides used to cultivate diverse commercial crops. However, their high toxicity and persistence in the soil also brought troublesome environmental problems. For screening microbes that can effectively degrade imidazolinone compounds, Chen et al. leverage multiple microfluidic approaches for chemotaxis screening of soil microbiota, as chemotaxis phenotype has been reported to correlate with degradation efficiency of pollutants. In their work, enrichment of microbial cells with higher chemotaxis performance toward imidazolinones is carried out on a specific SlipChip that can generate chemical gradients (Shen et al., 2014). Microbial cells after chemotaxis enrichment are then isolated and cultured using the MSP method. The population structure of the microbial consortium after chemotaxis enrichment changes significantly. More importantly, the consortium degradation performance increases by approximately 10%. The obtained strains after MSP isolation also show a broader imidazolinones degradation spectrum compared with previously reported strains (Chen et al., 2019).

In addition to screening living cells, microfluidics is also competent in screening functional genes of both culturable and unculturable taxa in a microbiome. This is realized by constructing a metagenomic library of the microbiome, and then screening each gene in the library with droplet microfluidics. The workflow of creating a metagenomic library involves microbiota sampling, DNA extraction, fragmentation, inserting DNA fragments into plasmid vectors, and transforming plasmids to host microbial cells for gene expression. The subsequent screening of the metagenomic library can be flexibly realized by using microfluidic FADS (Colin et al., 2015), flow cytometry-enabled droplet sorting (Figure 5C; Ma et al., 2021), or even fluorescence imaging assay (Hosokawa et al., 2015). Based on this approach, the microbiome sampled from soils and vanilla pods are screened for new hydrolases of sulfate monoesters and phosphotriesters. Six and eight hits out of 1,250,000 clones for sulfatase and phosphotriesterase are identified, respectively. Most of them are rare enzymes that possess promiscuous activities. Further studies show that most hits cannot be inferred from metagenomic information because these functions have never been ascribed to similar sequences (Colin et al., 2015). In another work, the microbiome sampled from running water is screened for novel esterase. Four gene types corresponding to the esterase function are found in 1 million clones, and one of them belongs to the same family as serine hydrolases, named Est WY. Further analysis shows that Est WY has no identical representative proteins in the NCBI database (Ma et al., 2021).

The achievements mentioned above collectively implied the practical potential of microfluidics in the biofuel, bioindustry, and bioremediation areas. However, applying microfluidics for mining more extensive biological resources is still facing some challenges. For the scenarios in which particular fluorogenic substrates need to be designed to indicate metabolic or enzymatic activities, the leakage problem should be considered simultaneously. This dramatically increases the execution difficulty (Najah et al., 2013). Furthermore, the use of fluorogenic substrates may also bring other problems. For example, microbial hits that have a higher affinity to the fluorogenic substrate analogs but a lower affinity to desired substrates may be sorted, resulting in missing the real valuable hits (Tauzin et al., 2020). These problems may be solved from the perspective of biochemistry or chemical engineering. For example, the invention of universal indicator molecules with finer droplet retention performance; or the development of novel surfactants that can better limit the escape of different molecules in droplets. Meanwhile, we look forward to a higher level of technical integration or innovation that will keep evolving the microfluidic technology, thus improving our ability to access more valuable enzymes and microorganisms.

### Screening of health-related phenotypes

Mucin-degradation is one of the most concerned health-related phenotypes in the human gut microbiome, as it is considered highly correlated with inflammatory bowel diseases (IBD). Cataloging microbes with mucin-degrading ability as comprehensively as possible is fundamental to understanding IBD’s pathogenic mechanism, which is also the basis of making proper prophylactic and therapeutic schemes. Recently, microfluidic technologies have been used to investigate the mucin-degrading populations in the gut microbiome. Related works help us gain some insights into the characteristics of interactions between these populations and their hosts. In one study, a Raman-activated single-cell sorting microfluidic system is developed. Mucin is supplied as the sole carbon source for culturing, while D_2_O isotope is used to label living microbial cells (Lee et al., 2021). Microbiota from the mouse colon is screened and then metagenomically analyzed. Results show that mucin-degrading microbes constitute around 27.4% of the microbiota, while they are mainly genus *Bacteroides* that belong to an uncultured family *Muribaculaceae*. Metagenome-assembled genomes of the sorted cells are searched for the presence of enzymes involved in mucin degradation. The result revealed that 84% of these genomes encode at least one enzyme that can degrade O-glycans, a kind of glycan decoration on the mucin molecule (Lee et al., 2019). In another work, a flow cytometry-enabled droplet sorting method is adopted to screen the mucin-degrading population in the human gut microbiota sampled from distal ileum mucosa. The genus *Bacteroides* is recognized again to be responsible for mucin degradation. Thereinto, the species *Bacteroides vulgatus* and *Bacteroides plebeius* are more likely linked to IBD. Moreover, a new member of ganglioside degrading enzymes, Uhgb_G123, is identified. The prevalence of the genes encoding this enzyme in IBD patients implied the role of Uhgb_G123 in the inflammatory process (Tauzin et al., 2020). These findings offer us many clues about potential IBD therapeutic targets. However, the mechanism of IBD is complex, and bacterial cells belonging to different taxa may also be involved. Mutual interactions between these cells, as well as their interactions with the host, may play a vital role in the occurrence and development of the diseases. Utilizing diverse microfluidic approaches to study the role of the gut microbiome on IBD from different perspectives will help us better elaborate the mechanism.

On the other hand, curing IBD is still a big challenge so far. Crohn’s disease and ulcerative colitis are two typical chronic IBD, which may cause severe symptoms or even life-threatening complications. As of now, there is no known effective cure for these diseases. Fecal transplantation has shown gratifying results for treating recurrent *Clostridium difficile* colitis and it is considered a hopeful option to cure IBD. However, the medical use of therapeutics should be cautious enough, which means that the quality of the fecal microorganisms sampled from donors should be strictly controlled. Antibiotic resistance of microbiota is an important quality indicator of a fecal sample. Thus, examining antibiotic-resistant species in fecal microbiota becomes a critical step. Droplet microfluidics can provide a more accurate and rapid way in this respect. By encapsulating single bacterial cells in droplets together with antibiotic drugs, the resistance phenotype can be determined by examining bacterial growth in the droplets within several hours (Boedicker et al., 2008; Eun et al., 2011). The examination can be reported by either metabolic-induced fluorescent variation or cell proliferation-based light scattering change (Liu et al., 2016; Kaushik et al., 2017). Meanwhile, a droplet pool with a broad antibiotic gradient can be built up very quickly, and the growth of bacterial cells under different antibiotic concentrations can be detected simultaneously, which enables faster and more precise detection of minimum inhibitory concentration (Scheler et al., 2017, 2020; Kao et al., 2020). A recent work by Watterson et al. demonstrates how to screen antibiotic-resistant members in the fecal transplant microbiome using droplet microfluidics. They identify up to 21 important antibiotic-resistant microorganisms that were missed by traditional plate-based screening (Watterson et al., 2020). This finding reminds us of the insufficiency of current quality control of donor fecal samples and the potential health risks for large-scale fecal transplantation applications, implying the urgency to develop more accurate screening technologies.

### Cell–cell interactions and microbial ecology

On the other hand, microfluidics is also promising for studying the microbiome at the population level, because large-scale cell–cell interactions can be investigated in a more economical, compact, and high-throughput manner. The feasibility of using microfluidics for elucidating microbial interactions, such as competition, mutualism, and altruism, has been demonstrated elsewhere. Either pairwise or higher-order interactions in a microbial consortium can be inferred with the same efficiency, because microfluidics enables the simultaneous generation of a large number of parallel experimental groups that can cover all the possible combinations of cell–cell interaction situations with enough repeats. In a simplified case, a synthetic symbiotic pair composed of two auxotroph bacterial strains is constructed. The growth of each strain depends on the amino acid secreted by the other strain. This consortium is randomly dispersed into hundreds of droplets to form subpopulations composed of different population compositions and initial cell numbers. Culturing results show that only if the subpopulations that contain both strains can flourish (Park et al., 2011; Hsu et al., 2019). The same method can also infer other pairwise interactions like inhibition or competition. However, interrogating higher-order interactions need to integrate additional techniques. Hus et al. combine fluorescence microscopy with computer vision techniques to determine the abundance of multiple strains in hundreds to thousands of droplets. This method is used to identify higher-order interactions that occur in synthetic three-member consortia. Moreover, environmental factors like nutrition (amino acids or carbon sources) or perturbations (antibiotics) are incorporated into the system. It was found that the microbial interaction network may change from cooperative to competitive following the change in nutrition composition (Hsu et al., 2019). Beyond synthetic consortia, microfluidics is also proven competent in characterizing cell–cell interaction in a natural microbial community, for example, discovering compositions that promote or suppress the growth of an interested strain. This is especially useful for industrial or medical applications, such as developing probiotic formulas to cure chronic infectious diseases, or developing plant symbiotic bacterial agents to improve agricultural production. One such case is combining gel microdroplet and microdroplet to construct a group of co-culture populations composed of environmental microbiota and a candidate biofuel producer, *Chlorella sorokiniana*. Flow cytometry is utilized to recover the populations in which the growth of *Chlorella sorokiniana* is significantly promoted. A collection of bacteria that may promote the growth of *Chlorella sorokiniana* was identified. One species, *Pseudomonas* spp., was isolated, and its growth-enhancing ability was confirmed (Ohan et al., 2019). Nutrition composition may have a profound impact on this kind of growth-promoting interaction. And it is a big challenge to identify a microbial species or consortia that robustly benefit a target microbe under different nutrition conditions or even under the perturbation of other species. Kehe et al. develop a microfluidic massively parallel screening method to identify microbial compositions that facilitate the proliferation of *Herbaspirillum frisingense*, a model plant symbiont. Both suppressive and facilitative effects were observed under different carbon source conditions when screening almost 10,000 combinations of 14 species isolated from soil microbiota. Among all those combinations, two bacterial compositions that strongly facilitate the proliferation of *Herbaspirillum frisingense*, regardless of the carbon source type and community topology, were identified (Kehe et al., 2019).

Some microbial consortia exist in the form of biofilm. This usually happens when microbes inhabit extreme environments, such as the oral cavity. The oral cavity environment is featured by frequent fluid flushing, extreme temperature and oxygen fluctuation, wide nutrition variation, etc. (Kolenbrander et al., 2010). Most oral microbes form a multispecies biofilm to withstand this adverse environment. Understanding the cell–cell interactions in oral biofilm is essential for uncovering the effect of the oral microbiome on human health. Conventionally, *in vitro* models of the oral biofilm are built with flow cell devices (Foster and Kolenbrander, 2004). But this approach is incompetent to study biofilm dynamics for an extended period (Nance et al., 2013). Microfluidics is proven to sustain the viability of biofilm in long-term experiments. Meanwhile, it can emulate the oral environment better by turning fluid flushing parameters over a sizable hydrodynamic range. Moreover, many advanced biofilm characterization techniques can be flexibly integrated into microfluidic devices. For example, optical or high-resolution microscopy can be integrated to monitor the morphology and composition of the biofilm, and electrodes can be integrated for electrochemical studies of the biofilm (Pousti et al., 2018). A dental plaque biofilm system has been constructed based on a microfluidic approach in a high-throughput manner. A confocal laser scanning microscope has been integrated to quantify the effect of antimicrobials on biofilm (Nance et al., 2013; Samarian et al., 2014). Besides, a microfluidic device coated with a fluorescent nanoparticle-based sensor for real-time detection of pH change in oral biofilm has been developed. The classic Stephan curve, which describes the acidification and recovery of the oral cavity environment followed by sucrose or glucose addition, was reproduced in this device (Gashti et al., 2016). Lam et al. study the bacterial colonization and dental biofilm formation processes systematically with a microfluidic “artificial teeth” platform. This platform is composed of 128 independently controlled culture chambers. For each of these chambers, the dynamical regulation of bacterial cell loading, nutrition supply, metabolic waste flushing, and oxygen level maintenance is achievable. An inverted fluorescence microscope is used for real-time monitoring of the features of biofilm, including spatial arrangement of bacterial species, distribution of live and dead cells, cell adhesion mediated by carbon source, the influence of oxygen on bacterial composition, etc. (Lam et al., 2016). Many interesting cell–cell interaction phenomena in the biofilm were revealed in this work. For instance, *Streptococci* can enhance the adhesion capability of *Fusobacterium nucleatum* with the presence of sucrose, which may reflect a potential co-aggregate relationship between these two bacteria. In addition, *Fusobacterium nucleatum* showed enhanced tolerance to the aerobic condition when co-culturing with other species in biofilm. As *Fusobacterium nucleatum* is located in the inner biofilm region, it is demonstrated that oxygen was consumed by the outer species in biofilm, thus forming a hypoxic zone inside the biofilm.

These cases mentioned above have shown how microfluidics enables the integration of complex environmental factors into the study of cell–cell interaction. Apparently, this will help us better simulate the microbial interaction networks in their natural habitat, further leading to our better understanding of microbial ecology.

### Host-microbiome interaction

Besides cell–cell interactions within a microbiota, the interplay between microbiota and the host is considered another important issue, especially in the studies of the human microbiome, because host-microbiome interactions are proven closely related to human health and disease. Microfluidic organ-on-a-chip (OoC) systems are emerging as an advanced *in vitro* model to better recapitulate the *in vivo* physiological properties of human tissues, thus increasing attention is being paid to studying host-microbiome interactions in OoC systems (Park et al., 2017; Bein et al., 2018). Among diverse OoC that mimic different organs, the gut-on-chip (GoC) is most exploited for host-microbiome interaction research (Tan and Toh, 2020; Xiang et al., 2020). GoC enables the integration of complex biophysical stimuli like peristalsis and fluid shearing to better simulate the gastrointestinal environment. These biophysical parameters are shown to be critical for developing *in vivo* gastrointestinal cell types and tissue morphologies, as well as reproducing the physical interaction between host and bacterial cells (Puschhof et al., 2021). These are all impossible to realize in other *in vitro* models, such as transwell or organoid. Several recently published review articles have systematically summarized the progress of using GoC in studying host-microbiome interactions. One progress is using stem cells instead of immortalized cell lines as the cell source to construct GoC. GoC with human pluripotent stem cells (hiPSCs) or adult stem cells (ASCs) is considered to better recapitulate the genetic background of healthy people. GoC with biopsy-derived cells is thought to better resemble a patient-specific genetic background (Siwczak et al., 2021). Another progress is the development of multi-organ-on-a-chip (MoC) systems (Ashammakhi et al., 2020). MoC allows us to conduct researches that were not possible before. For example, MoC composed of GoC and brain-on-chip enables us to emulate the gut-brain axis. Thus, how the microbiome affects our brain, or even causes the mental disease can be investigated *in vitro* (Moysidou and Owens, 2021). In addition, MoC composed of GoC and liver enables us to simulate the gut–liver axis. This MoC can be used to study the microbiome’s effect on drug metabolism, thereby facilitating drug testing and safety assessment (De Filippis et al., 2021). Besides the aforementioned applications, GoC has been shown to conduct researches that relate to many other interactions between the gut and the microbiome, such as pathogen infection and gut inflammation, food digestion and metabolism, etc. (Steinway et al., 2020; Garcia-Gutierrez and Cotter, 2021). In addition to GoC, many other OoC systems have been constructed for different research purposes, but they were barely used to study host-microbiome interactions. Further exploration of these OoC systems for a more comprehensive analysis of human–microbiome interactions is expected.

## Summary and outlook

Microfluidics has been increasingly applied to study the microbiome in the past decade. Especially in recent years, there has been a burst of using microfluidic technologies to solve challenging tasks in microbiome research. Indeed, droplet microfluidics appears as a promising approach with diverse applications in studying the microbiome because of its unique characteristics, such as high throughput, low cost, versatility, flexibility, etc. Relevant achievements include the more comprehensive cultivation of widespread species, enhanced capability of culturing low-abundance species, increased possibilities to find new taxa, and more efficient detection and recovery of rare phenotypes or targeted species, etc. However, the relatively complex emulsion chemical system limits the broader application of droplet microfluidics. For example, the instability of droplets caused by the addition of certain reagents or cross-contamination between droplets caused by the leakage of small molecules. Microfluidic microwell array technology can help to address some of these problems, where physical barriers for cell compartments can be constructed by impermeable solid walls. Therefore, the side effects caused by droplet instability or metabolic product leakage can be neglected, but such improvement concurrently results in a significantly compromised throughput. Alternatively, the lower throughput can be rescued by a robotic handling machine to increase the microwell array processing throughput. But it requires a series of technical innovations such as developing automated monitoring, transferring, and maintaining methods for densely arranged samples with ultra-small volumes.

There is also increasing interest in applying microfluidics for promoting omics-based microbiome research. Omics-based approaches include genome profiling by DNA sequencing, transcriptome profiling by RNA sequencing, proteome profiling by protein detecting, etc. Microfluidics greatly enhanced the resolution and precision of these approaches through their outstanding high-throughput single-cell handling ability (Liu and Walther-Antonio, 2017). Through encapsulating single microbial cells into droplets, whole genome amplification (WGS) can be conducted to form single-amplified genomes (SAGs). Each SAG corresponds to a partial genome of a single cell originating from a single droplet. By assembling multiple SAGs of the same species, a high-quality genome with a low contamination rate and a high coverage rate is accessible (Hosokawa et al., 2017). Recent work has shown that strain-level classification of the human gut microbiome is achievable when integrating droplet-specific barcodes and advanced bioinformatic algorithms (Zheng et al., 2022). Microfluidics was also adopted to minimize the bias and contamination of bacterial single-cell whole transcriptome amplification (WTA). It was shown that high-quality gene expression data could be obtained even with femtograms to picograms of RNA from a single bacterial cell (Liu et al., 2019). Recent progress on single-cell RNA sequencing in barcoded droplets has shown previous undetected cell state in a genomic identical *Bacillus subtilis* population, a small number of cells process a transcriptomic signature indicative of sporulation (McNulty et al., 2021). Microfluidics can be utilized to infer single-cell proteomic when integrating with mass spectrometry, but current applications are limited to mammalian cells (Li et al., 2018; Gebreyesus et al., 2022). The development of related fields may largely rely on the further improvement of mass spectrometry sensitivity and detection limitation, which is essential for detecting trace amounts of protein in a single bacterial cell. Beyond these independent omics-based approaches, microfluidics has also gained intention as a powerful multi-omics technique (Prakadan et al., 2017; Lamanna et al., 2020; Dimitriu et al., 2022). However, the application of microfluidic-based multi-omics tools is limited to mammalian cell-related researches. Although transforming those microfluidic tools to accommodate microbial cell-related applications is technically challenging, we are still faithful in the future of this area, and we believe it will greatly promote the development of the microbiome researches.

Although microfluidic technologies have great potential in promoting the microbiome research, most of them are still in their infancy, because widespread applications in different laboratories and commercial R&D departments still face critical obstacles. For example, the mass production of specific microfluidic chips with modulated parameters is still high-cost and time-consuming. Thus, one must gain enough skills and experience in microfluidic designing, testing, and optimizing the devices and protocols, which tremendously reduces the accessibility of the technology. Fortunately, professionals in the microfluidic field are putting more effort into standardizing diverse microfluidic toolkits to satisfy the needs of various applications (Chin et al., 2012; Volpatti and Yetisen, 2014; Dekker et al., 2018; Reyes et al., 2021). These toolkits, combined with fast-growing artificial intelligence and automation technologies, could quickly pave a broad way for universal microfluidic applications in microbiome research.

## Author contributions

YY and SH conceptualized the topic and contents of the manuscript. YY and HW wrote and formatted the texts. SL, HC, XL, ZM, XS, and LZ provide expert opinions on the manuscript. All authors contributed to the article and approved the submitted version.

## Funding

This work was supported by the National Key Research and Development Program of China (2020YFA0908803), National Natural Science Foundation of China (31971350 and 32100032), Basic and Applied Basic Research Foundation of Guangdong Province (2020A1515110846), and Strategic Priority Research Program of the Chinese Academy of Sciences (XDPB17 and XDPB18).

## Conflict of interest

The authors declare that the research was conducted in the absence of any commercial or financial relationships that could be construed as a potential conflict of interest.

## Publisher’s note

All claims expressed in this article are solely those of the authors and do not necessarily represent those of their affiliated organizations, or those of the publisher, the editors and the reviewers. Any product that may be evaluated in this article, or claim that may be made by its manufacturer, is not guaranteed or endorsed by the publisher.
