# Corrigendum: Emerging microfluidic technologies for microbiome research

**DOI:** 10.3389/fmicb.2022.1038682

**Published:** 2022-09-26

**Authors:** Yue Yu, Hui Wen, Sihong Li, Haojie Cao, Xuefei Li, Zhixin Ma, Xiaoyi She, Lei Zhou, Shuqiang Huang

**Affiliations:** ^1^CAS Key Laboratory of Quantitative Engineering Biology, Guangdong Provincial Key Laboratory of Synthetic Genomics, Shenzhen Institute of Synthetic Biology, Shenzhen Institutes of Advanced Technology, Chinese Academy of Sciences, Shenzhen, China; ^2^Faculty of Synthetic Biology, Shenzhen Institute of Advanced Technology, Chinese Academy of Sciences, Shenzhen, China; ^3^University of Chinese Academy of Sciences, Beijing, China

**Keywords:** microfluidics, microbiome, microbial cultivation, phenotype screening, technologies integration

In the published article, there was an error in the Funding statement. [The funding statement for the National Key Research and Development Program of China (2020YFA0908803) was incorrectly listed at the end of the paragraph]. The correct Funding statement appears below.

## Funding

This work was supported by the National Key Research and Development Program of China (2020YFA0908803), National Natural Science Foundation of China (31971350 and 32100032), Basic and Applied Basic Research Foundation of Guangdong Province (2020A1515110846), and Strategic Priority Research Program of the Chinese Academy of Sciences (XDPB17 and XDPB18).

The authors apologize for this error and state that this does not change the scientific conclusions of the article in any way. The original article has been updated.

## Publisher's note

All claims expressed in this article are solely those of the authors and do not necessarily represent those of their affiliated organizations, or those of the publisher, the editors and the reviewers. Any product that may be evaluated in this article, or claim that may be made by its manufacturer, is not guaranteed or endorsed by the publisher.

